# Physiological and Optical Alterations Precede the Appearance of Cataracts in Cx46fs380 Mice

**DOI:** 10.1167/iovs.17-21684

**Published:** 2017-08

**Authors:** Peter J. Minogue, Junyuan Gao, Rebecca K. Zoltoski, Layne A. Novak, Richard T. Mathias, Eric C. Beyer, Viviana M. Berthoud

**Affiliations:** 1 Department of Pediatrics, University of Chicago, Chicago, Illinois, United States; 2 Department of Physiology and Biophysics, Stony Brook University, Stony Brook, New York, United States; 3 Illinois College of Optometry, Chicago, Illinois, United States

**Keywords:** gap junction coupling, refractive properties, connexin mutant, cataractogenesis

## Abstract

**Purpose:**

Cx46fs380 mice model a human autosomal-dominant cataract caused by a mutant lens connexin46, Cx46. Lenses from Cx46fs380 mice develop cataracts that are first observed at ∼2 months in homozygotes and at ≥4 months in heterozygotes. The present studies were conducted to determine whether Cx46fs380 mouse lenses exhibited abnormalities before there are detectable cataracts.

**Methods:**

Lenses from wild-type and Cx46fs380 mice were studied at 1 to 3 months of age. Connexin levels were determined by immunoblotting. Gap junctional coupling was calculated from intracellular impedance studies of intact lenses. Optical quality and refractive properties were assessed by laser scanning and by photographing a 200-mesh electron microscopy grid through wild-type and Cx46fs380 mouse lenses.

**Results:**

Connexin46 and connexin50 levels were severely reduced in mutant lenses. Gap junctional coupling was decreased in differentiating and mature fibers from Cx46fs380 lenses; in homozygotes, the mature fibers had no detectable coupling. Homozygous lenses were slightly smaller and had reduced focal lengths. Heterozygous and homozygous lenses significantly distorted the electron microscopy grid pattern as compared with wild-type lenses.

**Conclusions:**

Before cataract appearance, Cx46fs380 lenses have decreased gap junctional conductance (at least in heterozygotes) and alterations in refractive properties (heterozygotes and homozygotes). The decreased focal distance of Cx46fs380 homozygous lenses is consistent with an increase in refractive index due to changes in cellular composition. These data suggest that Cx46fs380 lenses undergo a sequence of changes before the appearance of cataracts: low levels of connexins, decreased gap junction coupling, alterations in lens cell homeostasis, and changes in refractive index.

Congenital cataracts can result from mutations of several genes encoding major lens proteins including crystallins, transcription factors, and membrane proteins such as aquaporin0 and the gap junction proteins connexin46 (Cx46) and connexin50 (Cx50).^1,2^ To gain insights into the changes and mechanisms leading to cataract formation due to a connexin mutant, we generated mice that express Cx46fs380 from the locus of wild-type Cx46 to mimic one of the human cataract-associated lens connexin mutants.^3^ This mutant has a frameshift starting at amino acid 380 of Cx46,^4^ generating a diphenylalanine motif that leads to improper trafficking of the protein.^5^ These mice develop progressive cataracts, which are detected at 2 months in Cx46fs380 homozygotes and at ≥4 months in heterozygous mice.^3^ Although the lenses of these animals are transparent at 1 month of age, the levels of Cx46 are drastically decreased, and Cx50 levels show a milder reduction.^3^

Because gap junctions are crucial for the lens microcirculation system, the decrease in lens fiber connexins suggested that expression of Cx46fs380 may alter lens cell homeostasis and induce changes that affect optical quality before the detection of opacities. Therefore, we analyzed these aspects in Cx46fs380-expressing lenses.

## Materials and Methods

### Animals

Experiments were performed on Cx46fs380 mice, which were generated on a mixed 129/C57BL/6 genetic background but do not carry the CP49 mutation from the 129 mouse strain.^3^

All animal procedures were performed in accordance with the ARVO Statement for the Use of Animals in Ophthalmic and Vision Research and followed the Institutional Animal Care and Use Committee guidelines from the University of Chicago and Stony Brook University.

Animal ages are reported in months, based on 30-day months.

### Intracellular Impedance and Intracellular Resistivity

Mice were killed at approximately 2 months of age by peritoneal injection of pentobarbital (100 mg/kg of weight). The eyes were removed and placed in a Petri dish containing normal Tyrode's solution (137.7 mM NaCl, 2.3 mM NaOH, 5.4 mM KCl, 2 mM CaCl_2_, 1 mM MgCl_2_, 5 mM HEPES, 10 mM glucose, pH 7.4). The cornea and iris were removed, and the optic nerve was cut. The sclera was cut into four flaps from the posterior surface and pinned to a Sylgard-lined chamber, where the experimental protocol was implemented. All experiments were conducted on freshly dissected lenses. These experiments required several days to complete studies of all animals.

Intracellular impedance was measured by using intracellular microelectrodes filled with 3 M KCl with initial resistances of 1.5 to 2 MΩ as described in the study of Mathias et al.^6^ The frequency domain impedance data were curve fitted with an equivalent circuit model of the intact lens originally derived by Eisenberg et al.^7^ and modified by Mathias et al.^6^ The equivalent circuit is distributed in nature and depends on both the Fourier angular frequency (*jω S*^−1^) and the distance from the lens center (*r cm*).

\newcommand{\bialpha}{\boldsymbol{\alpha}}\newcommand{\bibeta}{\boldsymbol{\beta}}\newcommand{\bigamma}{\boldsymbol{\gamma}}\newcommand{\bidelta}{\boldsymbol{\delta}}\newcommand{\bivarepsilon}{\boldsymbol{\varepsilon}}\newcommand{\bizeta}{\boldsymbol{\zeta}}\newcommand{\bieta}{\boldsymbol{\eta}}\newcommand{\bitheta}{\boldsymbol{\theta}}\newcommand{\biiota}{\boldsymbol{\iota}}\newcommand{\bikappa}{\boldsymbol{\kappa}}\newcommand{\bilambda}{\boldsymbol{\lambda}}\newcommand{\bimu}{\boldsymbol{\mu}}\newcommand{\binu}{\boldsymbol{\nu}}\newcommand{\bixi}{\boldsymbol{\xi}}\newcommand{\biomicron}{\boldsymbol{\micron}}\newcommand{\bipi}{\boldsymbol{\pi}}\newcommand{\birho}{\boldsymbol{\rho}}\newcommand{\bisigma}{\boldsymbol{\sigma}}\newcommand{\bitau}{\boldsymbol{\tau}}\newcommand{\biupsilon}{\boldsymbol{\upsilon}}\newcommand{\biphi}{\boldsymbol{\phi}}\newcommand{\bichi}{\boldsymbol{\chi}}\newcommand{\bipsi}{\boldsymbol{\psi}}\newcommand{\biomega}{\boldsymbol{\omega}}\begin{equation}\tag{1}Z\left( r,j\omega  \right) = {Z_L}\left( {j\omega } \right) + {R_s}\left(r\right)\end{equation}

The first term in Equation 1 is the input impedance of the intact lens. It is given by:

\newcommand{\bialpha}{\boldsymbol{\alpha}}\newcommand{\bibeta}{\boldsymbol{\beta}}\newcommand{\bigamma}{\boldsymbol{\gamma}}\newcommand{\bidelta}{\boldsymbol{\delta}}\newcommand{\bivarepsilon}{\boldsymbol{\varepsilon}}\newcommand{\bizeta}{\boldsymbol{\zeta}}\newcommand{\bieta}{\boldsymbol{\eta}}\newcommand{\bitheta}{\boldsymbol{\theta}}\newcommand{\biiota}{\boldsymbol{\iota}}\newcommand{\bikappa}{\boldsymbol{\kappa}}\newcommand{\bilambda}{\boldsymbol{\lambda}}\newcommand{\bimu}{\boldsymbol{\mu}}\newcommand{\binu}{\boldsymbol{\nu}}\newcommand{\bixi}{\boldsymbol{\xi}}\newcommand{\biomicron}{\boldsymbol{\micron}}\newcommand{\bipi}{\boldsymbol{\pi}}\newcommand{\birho}{\boldsymbol{\rho}}\newcommand{\bisigma}{\boldsymbol{\sigma}}\newcommand{\bitau}{\boldsymbol{\tau}}\newcommand{\biupsilon}{\boldsymbol{\upsilon}}\newcommand{\biphi}{\boldsymbol{\phi}}\newcommand{\bichi}{\boldsymbol{\chi}}\newcommand{\bipsi}{\boldsymbol{\psi}}\newcommand{\biomega}{\boldsymbol{\omega}}\[{Z_L}\left( {j\omega } \right) = {1 \over {\pi {a^2}\left( {{Y_s}\left( {j\omega } \right) + {Y_e}\left( {j\omega } \right)} \right)}}\]

\newcommand{\bialpha}{\boldsymbol{\alpha}}\newcommand{\bibeta}{\boldsymbol{\beta}}\newcommand{\bigamma}{\boldsymbol{\gamma}}\newcommand{\bidelta}{\boldsymbol{\delta}}\newcommand{\bivarepsilon}{\boldsymbol{\varepsilon}}\newcommand{\bizeta}{\boldsymbol{\zeta}}\newcommand{\bieta}{\boldsymbol{\eta}}\newcommand{\bitheta}{\boldsymbol{\theta}}\newcommand{\biiota}{\boldsymbol{\iota}}\newcommand{\bikappa}{\boldsymbol{\kappa}}\newcommand{\bilambda}{\boldsymbol{\lambda}}\newcommand{\bimu}{\boldsymbol{\mu}}\newcommand{\binu}{\boldsymbol{\nu}}\newcommand{\bixi}{\boldsymbol{\xi}}\newcommand{\biomicron}{\boldsymbol{\micron}}\newcommand{\bipi}{\boldsymbol{\pi}}\newcommand{\birho}{\boldsymbol{\rho}}\newcommand{\bisigma}{\boldsymbol{\sigma}}\newcommand{\bitau}{\boldsymbol{\tau}}\newcommand{\biupsilon}{\boldsymbol{\upsilon}}\newcommand{\biphi}{\boldsymbol{\phi}}\newcommand{\bichi}{\boldsymbol{\chi}}\newcommand{\bipsi}{\boldsymbol{\psi}}\newcommand{\biomega}{\boldsymbol{\omega}}\begin{equation}\tag{2}{Y_S}\left( {j\omega } \right) = {G_s} + j\omega {C_s}\end{equation}

\newcommand{\bialpha}{\boldsymbol{\alpha}}\newcommand{\bibeta}{\boldsymbol{\beta}}\newcommand{\bigamma}{\boldsymbol{\gamma}}\newcommand{\bidelta}{\boldsymbol{\delta}}\newcommand{\bivarepsilon}{\boldsymbol{\varepsilon}}\newcommand{\bizeta}{\boldsymbol{\zeta}}\newcommand{\bieta}{\boldsymbol{\eta}}\newcommand{\bitheta}{\boldsymbol{\theta}}\newcommand{\biiota}{\boldsymbol{\iota}}\newcommand{\bikappa}{\boldsymbol{\kappa}}\newcommand{\bilambda}{\boldsymbol{\lambda}}\newcommand{\bimu}{\boldsymbol{\mu}}\newcommand{\binu}{\boldsymbol{\nu}}\newcommand{\bixi}{\boldsymbol{\xi}}\newcommand{\biomicron}{\boldsymbol{\micron}}\newcommand{\bipi}{\boldsymbol{\pi}}\newcommand{\birho}{\boldsymbol{\rho}}\newcommand{\bisigma}{\boldsymbol{\sigma}}\newcommand{\bitau}{\boldsymbol{\tau}}\newcommand{\biupsilon}{\boldsymbol{\upsilon}}\newcommand{\biphi}{\boldsymbol{\phi}}\newcommand{\bichi}{\boldsymbol{\chi}}\newcommand{\bipsi}{\boldsymbol{\psi}}\newcommand{\biomega}{\boldsymbol{\omega}}\[{Y_e}\left( {j\omega } \right) = {{\gamma (J\omega )} \over {{R_e}}}(\coth \gamma \left( {j\omega } \right)a - {1 \over {\gamma \left( {j\omega } \right)a}}),\gamma (j\omega ) = \sqrt {{R_e}{{{S_m}} \over {{V_T}}}({g_m} + j\omega {c_m})} \]

The parameters in Equation 2 represent the following: lens radius (cm) = *a*; surface cell membrane conductance (S/cm^2^) = *G_s_*; surface cell membrane capacitance (F/cm^2^) = *C_s_*; fiber cell membrane conductance (S/cm^2^) = *g_m_*; fiber cell membrane capacitance (F/cm^2^) = *c_m_*; surface of fiber cell membrane/unit volume tissue (cm^−1^) = *S_m_*/*V_T_* ; effective extracellular resistivity (Ω-cm) = *R_e_*.

The magnitude of the lens impedance is given by |*Z*(*r*, *jω*)| in Equation 1; the phase angle is given by *Phase = arctan*{*I_m_*(*Z*(*r*, *jω*)/*R_e_*(*Z*(*r*, *jω*)}. If a sinusoidal current of amplitude *I* (amps) and frequency *ω* is injected into a central fiber cell, the responding intracellular voltage would also be a sinusoid of frequency *ω* and amplitude *V*(*r*), where *r* is the distance from the lens center, and the peak in the induced sinusoidal voltage would lag behind the peak of the injected current. The magnitude of the impedance at frequency *ω* is *V*(*r*)/*I*, while the phase angle is the normalized time delay between the two peaks.

The second term in Equation 1 is the series resistance (*R_s_*(*r*) Ω), which is the high frequency asymptote of the magnitude of the lens input impedance. At high frequencies, the impedance of membranes becomes negligible (*Z_L_* (*jω*) → 0) and the lens behaves like a spherical conductor with a point source of current at its center, and its surface at ground potential. This model,^6^ was used to analyze *R_s_* data, and is given by:

\newcommand{\bialpha}{\boldsymbol{\alpha}}\newcommand{\bibeta}{\boldsymbol{\beta}}\newcommand{\bigamma}{\boldsymbol{\gamma}}\newcommand{\bidelta}{\boldsymbol{\delta}}\newcommand{\bivarepsilon}{\boldsymbol{\varepsilon}}\newcommand{\bizeta}{\boldsymbol{\zeta}}\newcommand{\bieta}{\boldsymbol{\eta}}\newcommand{\bitheta}{\boldsymbol{\theta}}\newcommand{\biiota}{\boldsymbol{\iota}}\newcommand{\bikappa}{\boldsymbol{\kappa}}\newcommand{\bilambda}{\boldsymbol{\lambda}}\newcommand{\bimu}{\boldsymbol{\mu}}\newcommand{\binu}{\boldsymbol{\nu}}\newcommand{\bixi}{\boldsymbol{\xi}}\newcommand{\biomicron}{\boldsymbol{\micron}}\newcommand{\bipi}{\boldsymbol{\pi}}\newcommand{\birho}{\boldsymbol{\rho}}\newcommand{\bisigma}{\boldsymbol{\sigma}}\newcommand{\bitau}{\boldsymbol{\tau}}\newcommand{\biupsilon}{\boldsymbol{\upsilon}}\newcommand{\biphi}{\boldsymbol{\phi}}\newcommand{\bichi}{\boldsymbol{\chi}}\newcommand{\bipsi}{\boldsymbol{\psi}}\newcommand{\biomega}{\boldsymbol{\omega}}\begin{equation}\tag{3}{R_s}\left( r \right) = \left\{ {\matrix{ {{{{R_{DF}}} \over {4\pi }}\left( {{1 \over r} - {1 \over a}} \right)\quad b \le r \le a} \cr {{{{R_{DF}}} \over {4\pi }}\left( {{1 \over b} - {1 \over a}} \right) + {{{R_{MF}}} \over {4\pi }}\left( {{1 \over r} - {1 \over b}} \right)\quad 0 \le r \le b} \cr } } \right.\end{equation}

The radial distance from the lens center is *r* (cm), the lens radius is *a* (cm), and the transition from differentiating fibers (DFs) to mature fibers (MFs) occurs at *r* = *b* (cm), where experience suggests that *b* = 0.85 *a*. The effective intracellular resistivity is not uniform and has the value *R_DF_* (Ω-cm) in DF and *R_MF_* (Ω-cm) in MF. The scaling resistivities in *R_s_* are more accurately given by the parallel resistivities for extracellular (*R_e_* Ω-cm) and intracellular current flow. Under normal physiological conditions, *R_e_* ≫ *R_DF,MF_*, so Equation 3 is presented in terms of *R_DF,MF_*. However, in some of the lenses studied here, gap junction coupling was very low, particularly in MF, in which case the best-fit value of *R_MF_* actually is limited to the value of *R_e_*.

### Immunoblotting

Whole lens homogenates from 2-month-old (59–61 days) mice were prepared in PBS, 4 mM EDTA, 2 mM phenylmethanesulfonyl fluoride, and cOmplete EDTA-free protease inhibitor cocktail (Roche Applied Science, Indianapolis, IN, USA) using a glass-glass homogenizer, sonicated, and subjected to immunoblotting by using anti-Cx46 intracellular loop and anti-Cx50 C-terminus antibodies as previously described.^8,9^ Equal loading and electrotransfer of the proteins was confirmed by Ponceau S staining of the membrane. Three independent experiments containing all genotypes were performed. The intensity of the bands was analyzed by densitometry using Adobe Photoshop (Adobe Systems, Inc., San Jose, CA, USA). Results are reported as percentages of the values determined from wild-type samples. Statistical significance was assessed by using Student's *t*-test.

### Determination of Lens Optical Quality

Mouse lenses from 1.4- to 1.8-month-old (43–54 days) littermates were subjected to helium neon laser scan analysis as described in Kuszak et al.^10^ to determine back vertex distance (BVD) and BVD variability. An average of 20 data points were collected from each lens. Data are presented as mean ± SEM; the number of animals used is indicated in parenthesis. SigmaPlot Systat Software (San Jose, CA, USA) was used to perform a 1-way ANOVA; differences between the genotypes with *P* < 0.05 were considered significant.

The refractive properties of lenses from 1-month-old (30–32 days) and 3.2-month-old (94–95 days) mice were evaluated by photographing them on top of a 200-mesh electron microscopy grid as previously performed by Shiels et al.^11^ The deformations of the grid pattern were evaluated by using the bUnwarp plug-in from ImageJ^12,13^ (http://imagej.nih.gov/ij/; provided in the public domain by the National Institutes of Health, Bethesda, MD, USA) and are reported as the average of the warping index ± SEM. Statistical significance was assessed by using Student's *t*-test.

### Determination of Lens Size

Lens size was ascertained in lenses from 1.6-month-old (49 days) and 6.4-month-old (191 days) mice as previously described by Shi et al.^14^ Preliminary experiments using lenses from male and female mice showed that lens size was not affected by sex in the Cx46fs380 mouse line. Therefore, data from male and female mouse lenses were combined. The equatorial and anteroposterior diameters were determined from photomicrographs by using ImageJ.^15^ In these images, each pixel represented ∼3 μm. The radii of curvature of the lens anterior and posterior surfaces were manually determined on printouts (at ×40 magnification) of the digital images by using a compass. These data are presented as the average ± SD. Statistical comparisons were performed by using Student's *t*-test.

## Results

### Expression of Cx46fs380 Altered Lens Electrophysiological Properties

**Figure 1:**
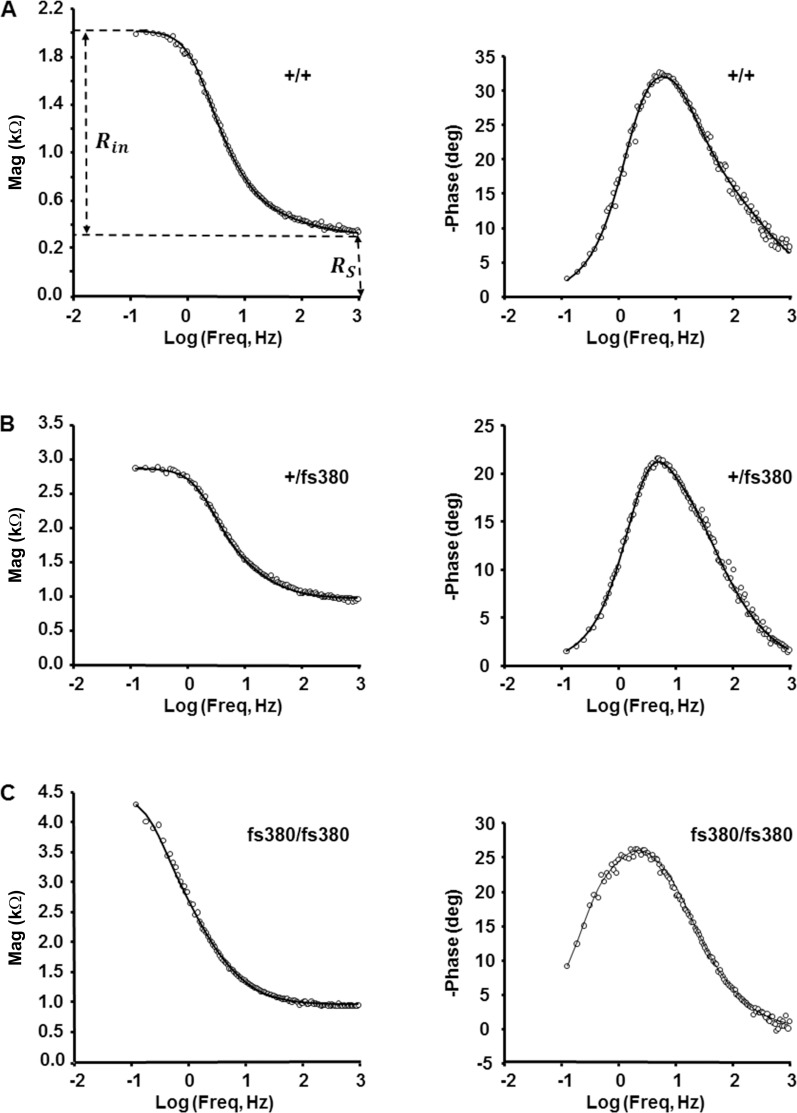
Cx46fs380 expression affects lens impedance. (A–C) Magnitude and phase angle of the impedance of intact lenses from wild-type (A), Cx46fs380 heterozygous, (B) and Cx46fs380 homozygous (C) mice recorded in differentiating fiber cells. The smooth curves are the best fits of Equations 1 and 2 to the data.

To assess the effect of expression of Cx46fs380 on lens gap junction conductance, we first needed to determine the series resistance at different distances from the center of the lens. Thus, we performed frequency domain impedance studies on freshly dissected intact mouse lenses. We studied lenses from 2-month-old mice (even though the homozygotes have cataracts at this age) to facilitate comparison with previous studies.^16,17^ The data were compared to a structurally based equivalent circuit model (Equations 1 and 2) describing the linear electrical properties of a spherically symmetric syncytial tissue with different membrane conductances for surface versus interior cells. At low frequencies, the magnitude of the impedance approaches the input resistance of the lens *R_in_* = *Z_L_*(0) (Equation 2) and the phase angle approaches zero. At high frequencies, the magnitude of the impedance asymptotes to the series resistance (*R_s_*(*r*) *k*Ω) and the phase angle approaches zero, since there is no time delay between voltage and current flow through a simple resistor. Figure 1 shows examples of the magnitude of the lens impedance (left panels) and phase angle (right panels) obtained at different distances from the center of the lens. The phase angle has been graphed as a positive angle, but it is actually a negative shift. The smooth curves are the best fits of the model in Equations 1 and 2. Impedance data could not be obtained all the way into the center of the lenses studied here, because the lens core is relatively hard and tends to clog the recording microelectrode tip, increasing its resistance and making the records unreliable; this problem was worst in homozygous lenses.

The phase angle of the impedance is much more sensitive than the magnitude to the various time constants that define the equivalent circuit, and it is also more sensitive to artifacts due to stray capacitances. If the electrode resistance becomes too high, the stray capacitances cannot be compensated and the data, particularly the phase data, become unreliable. Because the microelectrodes were frequently blocked when studying the homozygous lenses, we had only 5 successful impedance experiments on these lenses, compared to 17 on heterozygous and 18 on wild-type lenses. Even in some of the data we analyzed, such as the records shown in Figure 1C, there were indications of high frequency artifacts, because the phase data go through zero at high frequency.

Table 1 summarizes the results of curve fitting the equivalent circuit model of the lens described by Equations 1 and 2 to the frequency domain impedance data. The solution to these equations is based on the assumption that the effective intracellular resistivity is much smaller than the effective extracellular resistivity, a condition that is reversed for homozygous lenses and therefore, the model-dependent parameters are not presented for homozygous lenses.

**Table 1:**
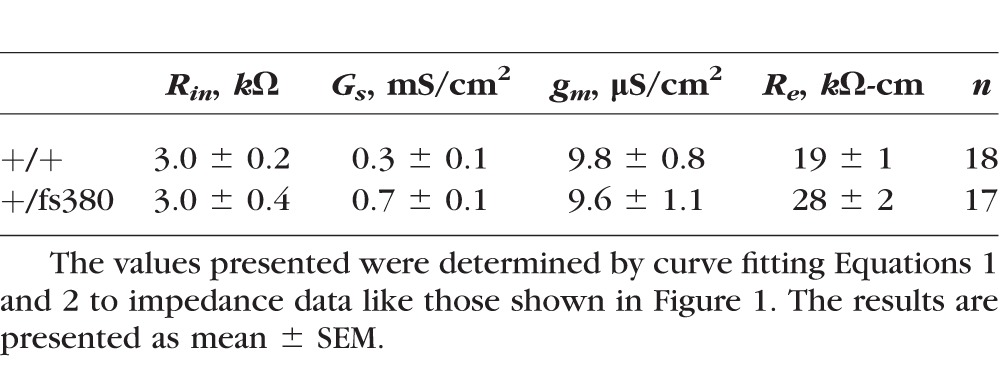
Estimated Equivalent Circuit Parameter Values for the Impedance Data of Wild-Type and Heterozygous Cx46fs380 Lenses

In the three types of lenses, the input resistance (*R_in_*, which is a model-independent measure of the overall effects of a number of more specific changes) did not change much, but it may have decreased somewhat in homozygous lenses where the estimated value was 2.6 ± 0.5 κΩ (*n* = 5). Surface cell membrane conductance (*G_s_*) increased from wild type to heterozygotes, whereas fiber cell membrane conductance (*g_m_*) did not change. If this trend could be extrapolated to homozygous lenses, then increased *G_s_* might account for the reduction in *R_in_*. The effective extracellular resistivity *R_e_* increased approximately 1.5-fold from wild type to heterozygotes.

**Figure 2:**
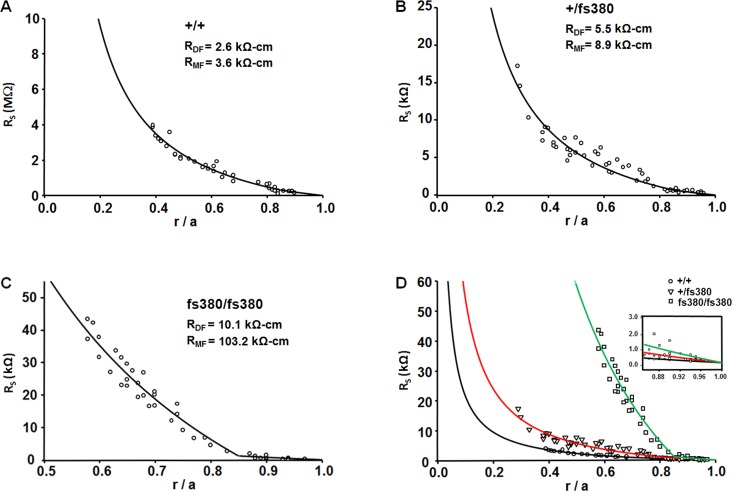
Gap junctional conductance is decreased in Cx46fs380 lenses. (A–C) Series resistance (R_s_) data graphed as a function of normalized radial distance from the lens center (r/a) recorded from wild-type (A), heterozygous mutant, (B) and homozygous mutant (C) lenses. The effective resistivities, R_DF_ and R_MF_, were determined by fitting Equation 3 to the data. (D) Comparison of the data from the three types of lenses. The inset shows an expanded view of the data in the differentiating fiber cells (r/a ≥ 0.85).

Once we obtained the series resistance from the frequency domain impedance studies at different distances from the lens center, the values were plotted against the relative radial distance at which they were obtained (*r*/*a*) (Fig. 2). Lenses from all three genotypes showed a change in slope in the radial dependence of the series resistance at *r* = *b*, but this was most evident in homozygous lenses (Fig. 2). The data were fitted by using Equation 3 (Fig. 2, smooth curves) with the assumption (based on previous studies in other lenses, where data can be obtained all the way into the center) that *R_MF_* is constant from *r* = 0 to *r* = *b*. The best-fit values of the effective intracellular resistivities in DF and MF cells (*R_DF_* and *R_MF_ k*Ω-*cm*) are included on the graphs. The corresponding values of *G_DF_* and *G_MF_* (the radial conductances per area of cell-to-cell contact due to gap junction coupling in DF and MF cells, respectively) are presented in Table 2.

**Table 2:**
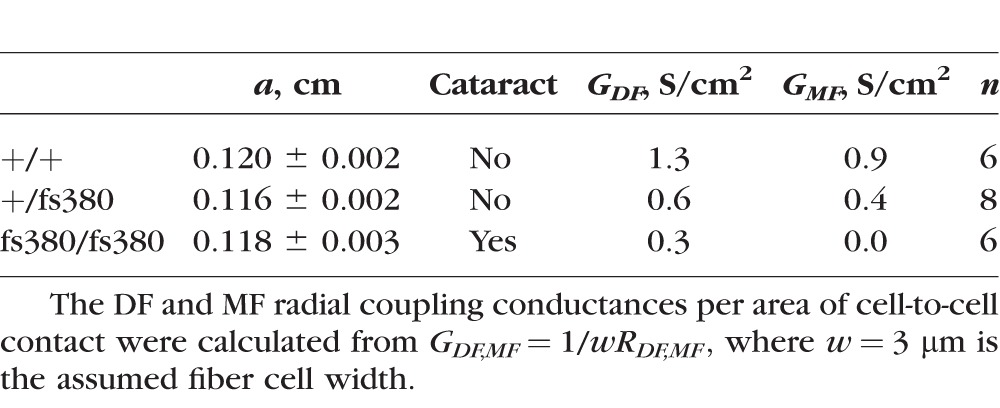
Cx46fs380 Fiber Cell Gap Junction Coupling Conductances

Both heterozygous and homozygous lenses showed decreased gap junctional conductance in DF and MF cells. In the MFs of homozygous Cx46fs380 lenses, the radial gradient in *R_s_* was so steep that the best-fit resistivity is limited by the parallel path for current flow through extracellular spaces. Thus, in these lenses, the MF gap junction coupling conductance was zero (to the resolution of our method).

### Levels of Lens Fiber Cell Connexins Are Reduced in Cx46fs380 Mice

**Figure 3:**
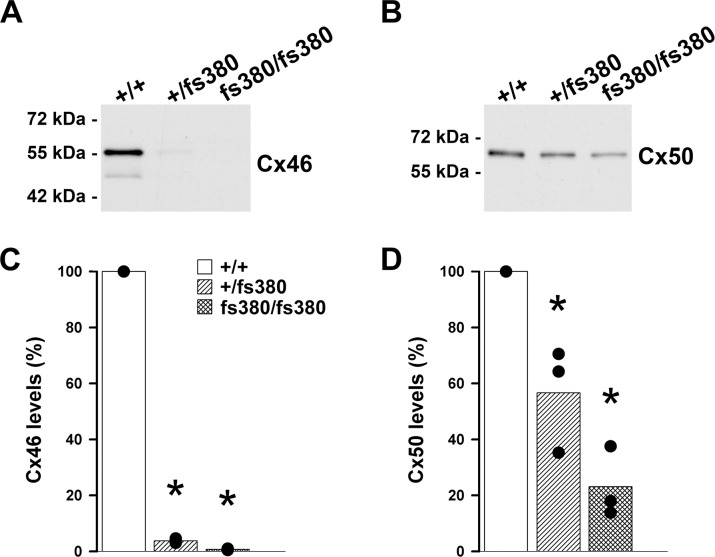
Levels of Cx46 and Cx50 are significantly decreased in Cx46fs380 lenses. (A, B) Immunoblots show the levels of immunoreactive Cx46 and Cx50 in total lens homogenates from 2-month-old wild-type (+/+) and Cx46fs380 heterozygous (+/fs380) and homozygous (fs380/fs380) mice. (C, D) Graphs show the densitometric values of the bands obtained in three independent experiments expressed as percentages of the values obtained in wild-type animals. Significant differences between wild-type and heterozygous Cx46fs380 or wild-type and homozygous Cx46fs380 lenses are indicated by asterisks (P < 0.05).

Our previous study has shown that connexin levels are decreased in Cx46fs380 heterozygous and homozygous lenses,^3^ but the extent of that reduction was not assessed at 2 months of age. To allow a valid comparison between the coupling conductance data and amounts of fiber lens connexins, we determined levels of Cx46 and Cx50 at this age. Levels of Cx46 and Cx50 were significantly reduced in both heterozygous and homozygous lenses of Cx46fs380 mice. On average, Cx46 was decreased to 3.8% (heterozygotes) and 0.7% (homozygotes), and Cx50 was reduced to 57% (heterozygotes) and 23% (homozygotes) of the wild-type values (Fig. 3).

### Expression of Cx46fs380 Affected Lens Optical Properties

**Figure 4:**
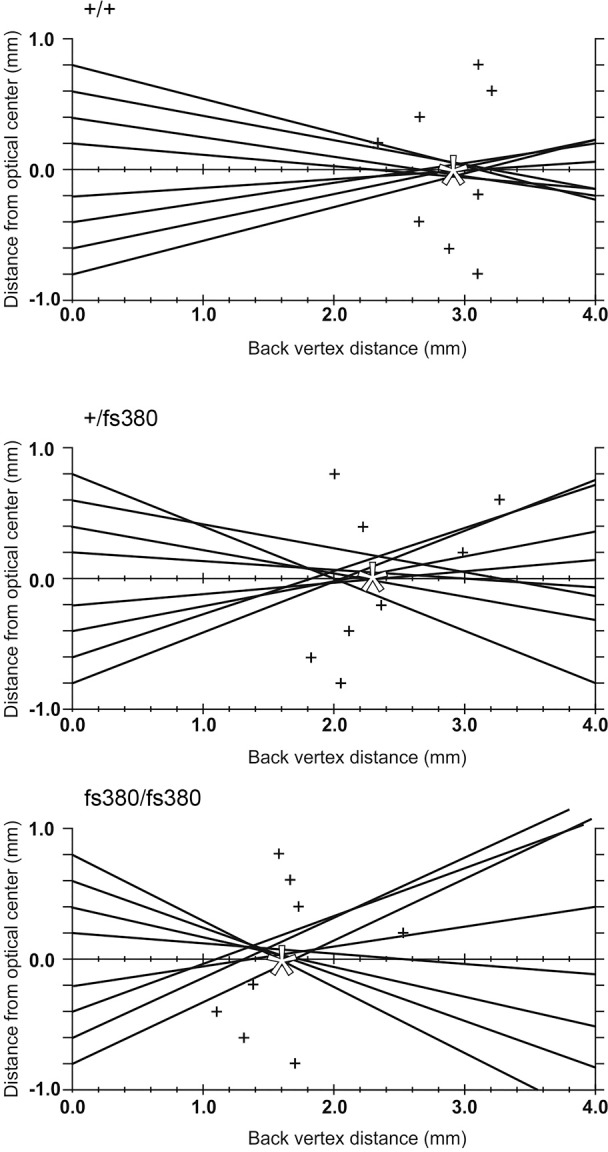
Examples of laser scan profiles from wild-type (+/+), and Cx46fs380 heterozygous (+/fs380) and homozygous (fs380/fs380) mouse lenses. The pathway of the laser beam (lines) and the data points (+) represent the focal point of each beam. The average BVD (white asterisk) was calculated from averaging the values of each of these data points, whereas the scatter of the points around the average is the variability in the BVD.

To determine whether Cx46fs380 lenses had other abnormalities before the appearance of cataracts, we used laser scanning to determine the focusing ability of mouse lenses from littermates of all genotypes at 1.4 to 1.8 months of age (Fig. 4). These experiments revealed a significant decrease in BVD in homozygous compared with wild-type lenses (1.6 ± 0.2 mm [*n* = 5] vs. 2.7 ± 0.2 mm [*n* = 5], *P* < 0.05). The BVD from heterozygous Cx46fs380 lenses (2.8 ± 0.3 mm [*n* = 5]) did not differ from wild-type lenses. The BVD variabilities (or SEM of individual points to the average BVD) did not differ among the genotypes. Their values were 0.37 ± 0.07, 0.32 ± 0.05, and 0.26 ± 0.06 in wild-type, heterozygous, and homozygous lenses, respectively.

**Figure 5:**
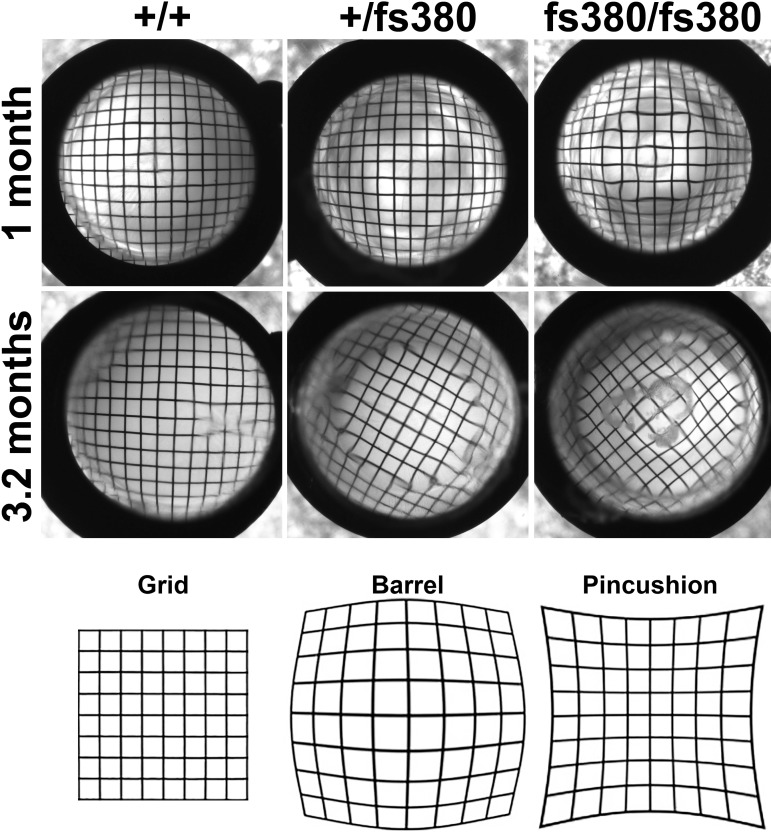
Expression of Cx46fs380 affects the refractive properties of the lens before the appearance of cataracts. Images were obtained by photographing lenses from 1- and 3.2-month-old wild-type (+/+) and Cx46fs380 heterozygous (+/fs380) and homozygous (fs380/fs380) mouse lenses against an electron microscopy grid. The pattern appears distorted at 3.2 months in heterozygotes and at both 1 and 3.2 months in homozygotes. Diagrams of the grid pattern and the barrel and pincushion deformations are shown at the bottom.

**Figure 6:**
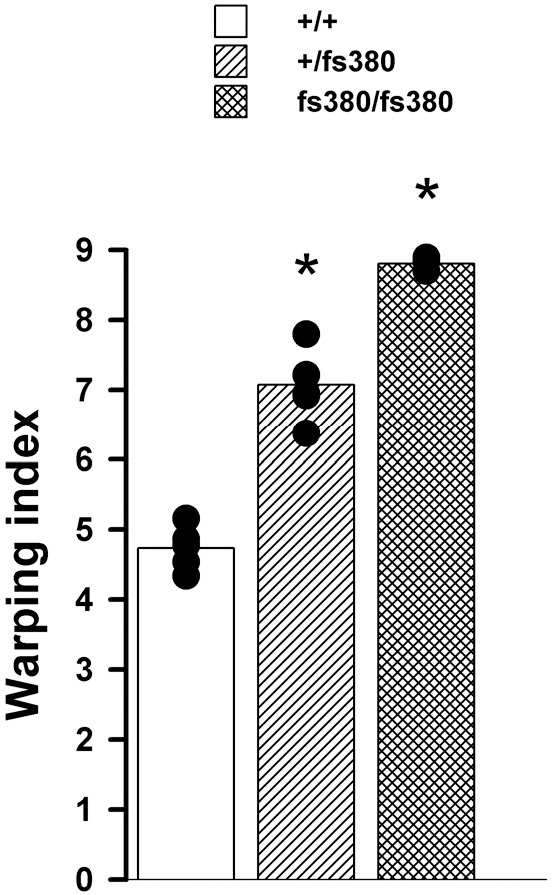
The warping index of the lens is altered in Cx46fs380 lenses before cataract appearance. The graph shows the warping index of wild-type (+/+), and mutant heterozygous (+/fs380) and homozygous (fs380/fs380) lenses from 1-month-old mice. The asterisks indicate significant differences between Cx46fs380 heterozygous or homozygous lenses versus wild-type lenses (P < 0.05).

We also analyzed the optical quality of Cx46fs380 lenses by photographing an electron microscopy grid through them. The grid pattern was magnified by wild-type lenses from 1- and 3.2-month-old animals to produce a barrel deformation, as expected for a wide angle lens (Fig. 5). The grid pattern was more distorted by Cx46fs380 heterozygous and homozygous lenses. At 1 month of age, heterozygous lenses produced a more pronounced barrel deformation than wild-type lenses, and homozygous Cx46fs380 lenses produced a pincushion deformation in the center surrounded by a barrel deformation (Fig. 5). A pincushion deformation is the opposite of a barrel deformation and is commonly seen in telephoto lenses (Fig. 5). At 3.2 months of age (an age at which homozygous lenses had cataracts), substantial distortions of the grid pattern were observed in Cx46fs380 heterozygous and homozygous lenses (Fig. 5). At this age, Cx46fs380 heterozygous and homozygous lenses deformed the grid to produce a peripheral barrel deformation surrounding a central pincushion deformation that affected a larger area as compared to 1-month-old homozygous lenses (Fig. 5). The pincushion area encompassed a greater area in 3.2-month-old homozygous than in 3.2-month-old heterozygous lenses (Fig. 5). To quantify the magnitude of the magnification and distortion before the appearance of cataracts, we determined the warping index at 1 month of age. The warping index was 4.73 ± 0.14 for wild-type lenses (*n* = 5), 7.08 ± 0.19 for heterozygous lenses (*n* = 6), and 8.81 ± 0.06 for homozygous lenses (*n* = 3). The values from heterozygotes and homozygotes were significantly different from wild type and from each other (Fig. 6). A slight deformation close to the edge of the lens was observed in all lenses, independent of genotype, and was not considered for the calculation of the warping index.

### Cx46fs380 Expression Had Minimal Effects on Lens Size and Shape

**Figure 7:**
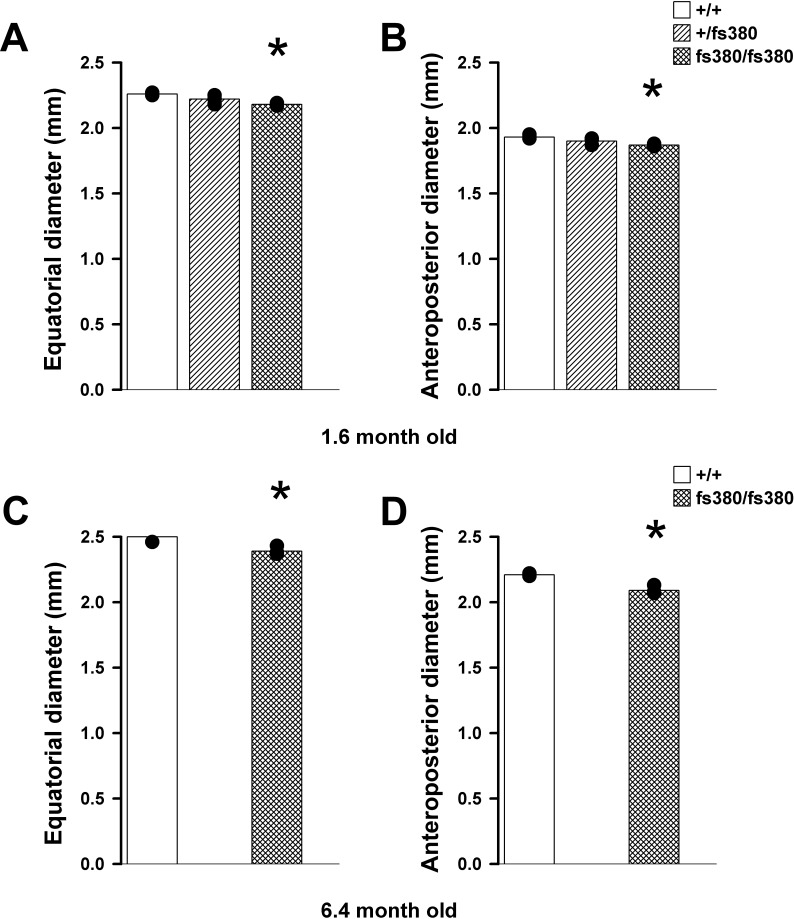
Homozygous Cx46fs380 mice have slightly smaller lenses. (A, B) The graphs show the equatorial (A) and anteroposterior (B) diameters of wild-type (+/+), heterozygous (+/fs380), and homozygous (fs380/fs380) lenses from 1.6-month-old mice. (C, D) The graphs show the equatorial (C) and anteroposterior (D) diameters of wild-type (+/+) and homozygous (fs380/fs380) lenses from 6.4-month-old mice.

Because the power of a lens (i.e., the reciprocal of its focal length or BVD) depends on lens thickness, the angle of curvature, and the index of refraction, we determined the lens equatorial and anteroposterior diameters and the radii of anterior and posterior surface curvatures of 1.6-month-old mice, an age comparable to that for which BVD had been determined. The equatorial and anteroposterior diameters of heterozygous lenses did not differ from wild-type lenses, but both values were slightly smaller (reduced by 3.1%–3.5%) in homozygous lenses (*P* < 0.05) (Fig. 7). At 6.4 months of age, the size of homozygous Cx46fs380 lenses continued to be smaller (by 4.4%–5.4%) than that of wild-type lenses (*P* < 0.05) (Fig. 7). The radius of the anterior surface curvature of homozygous lenses was slightly decreased as compared with that of wild-type lenses (1.16 ± 0.01 mm in wild-type lenses versus 1.13 ± 0.01 mm in homozygous lenses, *P* < 0.05; *n* = 3), but the posterior radius did not differ significantly from wild type (−1.14 ± 0.01 mm in wild-type lenses versus −1.12 ± 0.02 mm in homozygous lenses; *n* = 3). The anterior and posterior radii of curvature of heterozygous lenses were similar to those of wild-type lenses (anterior radius, 1.14 ± 0.03 mm; posterior radius, −1.13 ± 0.02 mm; *n* = 3).

## Discussion

We previously have shown that lenses from 1-month-old Cx46fs380 mice are transparent, but have reduced levels of both Cx46 and Cx50. At later ages, both heterozygous and homozygous mutant animals develop detectable cataracts.^3^ In this study, we showed that several optical and physiological alterations precede the appearance of Cx46fs380 cataracts, including increased warping index (in both heterozygotes and homozygotes), decreased focal distance and lens size (in homozygotes), and decreased gap junctional conductance (at least in heterozygotes).

Differences of lens shape and size can cause differences in optical properties. Before the appearance of cataracts, Cx46fs380 homozygous lenses were slightly smaller than the lenses of their wild-type littermates and had a minor decrease in the radius of the anterior surface curvature. These changes in lens size and shape would decrease BVD by ∼2% from wild-type values (i.e., from 2.7 to 2.64 mm). However, the BVD of homozygotes was 1.6 mm, which represents a 41% decrease in BVD. Since the changes in lens size and shape explain only 5.5% of the difference in BVD between wild-type and homozygous lenses, the decrease in BVD must result mostly from an increase in refractive index.

Alterations in the refractive properties of lenses have previously been reported for some mouse lines lacking expression of specific proteins, including *Lim2^Gt/Gt^*, which develop central pulverulent cataracts; CP49^−/−^, which show subtle opacification; and *Epha2^−/−^*, which do not develop cataracts.^11,14,18,19^ The changes in refractive properties in *Lim2^Gt/Gt^* and *Epha2^−/−^* lenses affect certain regions of the lens.^11,14^ In CP49^−/−^ lenses, both BVD and BVD variability are increased.^19^ In contrast, Cx46fs380 homozygous lenses had decreased BVD and had BVD variability similar to wild type.

Rodent lenses have a gradient of refractive index that can be approximated to a polynomial.^20,21^ This gradient is not completely smooth, as suggested by black gaps in laser scanning experiments and by determination of the refractive index profile using X-ray Talbot interferometry.^21,22^ In the murine lens, the profile has a kink at approximately 0.1 mm from the edge and a slight indentation at approximately 0.3 to 0.4 mm from the center of the lens.^21^ Heterozygous and homozygous Cx46fs380 lenses caused changes in deformation of the electron microscopy grid as compared with wild-type lenses; this implies changes in the refractive index, and therefore, of the internal composition of the fiber cells. In particular, the mixed distortions (pincushion surrounded by barrel deformation) observed in 3.2-month-old heterozygous lenses and ≥1-month-old homozygous lenses suggest that expression of Cx46fs380 leads to major alterations in the gradient of refractive index.

The distorted optics of Cx46fs380 lenses (when photographed over an electron microscopy grid) would be expected to be reflected in alterations in the angle of deflection of the laser beams after impacting the lens. Because the wild-type murine lens shows negative spherical aberration (i.e., the focal length is shorter for the more central rather than the marginal laser beams),^23^ and it produces a barrel deformation of the grid, it would be expected that a pincushion deformation corresponds to a decrease in negative spherical aberration. Indeed, the focal distance for some of the more central laser beams was longer than that of more marginal ones in Cx46fs380 homozygous lenses. Thus, it is possible that those beams impacted the lens at a position in which the lens induces a change (from barrel to pincushion) in the distortion of the grid. However, a similar phenomenon is observed in some of the heterozygous lenses, which cause a barrel deformation at this age. In addition, variability of BVD increases if the incident laser beam passes along a lens suture.^10^ Thus, a direct correlation between the data from the laser scanning experiments and the grid experiments cannot be drawn.

It is noteworthy that the type of grid deformation caused by heterozygous lenses depended on their age (i.e., barrel at 1 month of age versus mixed at 3.2 months of age). Since homozygous lenses already caused mixed deformation at 1 month of age, this suggests a slower time course for the alterations induced by Cx46fs380 expression in heterozygous lenses. We speculate that the sequence of alterations leading to cataract appearance in homozygous and heterozygous Cx46fs380 lenses is similar, but that heterozygous lenses lag behind homozygous lenses. Thus, the time course of the appearance of the pincushion deformation in Cx46fs380 lenses suggests that it is a precursor to cataracts.

Alterations in the gradient of refractive index can be induced by inhibiting the lens microcirculation system^24^ in which gap junction channels play a pivotal role. Because Cx46fs380 lenses have reduced levels of Cx46 and Cx50, lens gap junctional conductance was expected to be decreased. For Cx46fs380 homozygous lenses, *G_MF_* was unmeasurably small, whereas *G_DF_* was approximately 23% of the value in wild-type lenses. Previous studies of lens gap junction coupling suggest that both Cx50 and Cx46 form functional channels in DFs, but only Cx46 contributes functional channels in MFs (for review see Ref. 25). The gap junctional conductance values in Cx46fs380 homozygous lenses imply near total loss of Cx46 channels and partial loss of Cx50 channels. These conductance values directly correlated with the residual levels of Cx46 and Cx50 (0.71% and 23.1% of wild-type levels, respectively). In heterozygotes, the correlation was not as close: *G_DF_* was 46% and *G_MF_* was 31% of the wild-type values, while the remaining levels of Cx46 and Cx50 were 3.8% and 56.7% of the wild-type values, respectively. From the severe decrease in Cx46 levels, *G_MF_* would have been expected to be much smaller in heterozygous lenses. The reason for this discrepancy is not clear, but it is possible that Cx50 channels provide most of the remaining coupling conductance in heterozygous Cx46fs380 lenses. Because Cx46 and Cx50 levels are similarly reduced at 1 and 2 months of age, lens gap junctional conductance in 1-month-old Cx46fs380 heterozygous and homozygous mice is expected to be comparably decreased as at 2 months of age (i.e., before cataracts are evident in homozygotes). The transparency of the heterozygous lenses, at the time gap junction conductance was studied, implies that while a severe reduction in lens fiber cell coupling may eventually cause cataracts it does not happen immediately. However, uncoupling was associated with changes in lens optical quality.

The near absence of Cx46 levels suggests that the coupling conductances of Cx46fs380 lenses might be similar to those of Cx46KO lenses.^16^ This was most true for MFs: *G_MF_* was practically zero in both Cx46fs380 and Cx46KO homozygotes, while it was reduced to 31% in Cx46fs380 heterozygotes and 20% in Cx46KO heterozygotes. In DFs, Cx46fs380 lenses were more severely affected: *G_DF_* was decreased to 46% in Cx46fs380 heterozygotes versus 66% for Cx46KO and decreased to 23% in Cx46fs380 homozygotes versus 32% for Cx46KO. The more severe decrease in *G_DF_* in Cx46fs380 lenses is likely due to the decrease in Cx50 levels, unlike Cx46KO lenses, which have similar Cx50 levels to wild type.^16^ Variations of other genes may also contribute. Although both Cx46fs380 and Cx46KO mice were generated on a mixed 129/C57BL/6 background, the genetic backgrounds in these mouse colonies may have drifted in different directions over time. While CP49 is expressed in Cx46fs380 mice^3^ and absent in Cx46KO mice, its presence is unlikely to explain the physiological differences, because coupling conductance in CP49KO mice does not differ from that of wild-type mice.^26^

The changes in gap junction coupling conductance of Cx46fs380 lenses show some similarities to the age-dependent changes of wild-type lenses. In wild-type mice, gap junction coupling conductance decreases ∼2-fold at 6 months and ∼3.5-fold at 14 months as compared to 2-month-old lenses.^17^ Cx46fs380 heterozygous lenses showed a ∼2-fold decrease in coupling conductance relative to wild-type lenses, whereas Cx46fs380 homozygous lenses showed a 4.3-fold reduction in *G_DF_* and ∼28-fold reduction in *G_MF_*. These results would suggest that Cx46fs380 lenses are “aging” faster. However, in the aging study by Gao et al.,^17^ 14-month-old wild-type lenses did not develop cataracts, implying that aging involves compensatory changes in other systems to maintain lens transparency despite the decreased coupling.

The decrease in coupling conductance in Cx46fs380 lenses is expected to affect the lens microcirculation system. In cultured bovine lenses, inhibition of this system alters the gradient of refractive index and lens optical properties, including a myopic shift in vision that the authors consider analogous to the changes in optical power that precede cataract in presbyopic individuals.^24^ These results bear similarities with those found in Cx46fs380 homozygous lenses, which behaved myopically and caused mixed deformation of the grid before the detection of cataracts. It is not known whether human patients with the Cx46fs380 mutation suffer from myopia before cataract surgery, because bilateral cataract is usually present at birth or develops during infancy.^4^

Taken together, our results suggest that Cx46fs380 lenses progress through a sequence of changes before the appearance of cataracts. Expression of Cx46fs380 leads to low levels of connexins, resulting in decreased gap junctional coupling, which in turn alters the lens microcirculation system, affecting the internal composition of the lens fiber cells and leading to changes in the gradient of refractive index.
